# Breath-by-breath lung gas volume detection using GASMAS in a neonatal mannequin

**DOI:** 10.1038/s41390-025-04699-2

**Published:** 2026-01-10

**Authors:** Jurate Panaviene, Pranav Lanka, Konstantin Grygoryev, Andrea Pacheco, Sanathana Konugolu Venkata Sekar, Vicki Livingstone, Eugene M. Dempsey, Stefan Andersson-Engels

**Affiliations:** 1 INFANT Research Centre, University College Cork, Cork, Ireland; 2 Department of Neonatology, Cork University Maternity Hospital, Cork, Ireland; 3 Department of Paediatrics and Child Health, University College Cork, Cork, Ireland; 4 Irish Photonic Integration Centre, Tyndall National Institute, University College Cork Lee Maltings, Cork, Ireland; 5 School of Physics, University College Cork, Cork, Ireland

## Abstract

**Abstract:**

Monitoring lung gas volumes of ventilated infants is important. Gas in Scattering Media Absorption Spectroscopy (GASMAS) can estimate gas volume inside tissues by measuring oxygen absorption. We hypothesized that GASMAS can detect different tidal volumes (TV) delivered to mechanically ventilated lungs in a neonatal mannequin model breath-by-breath.

**Methods:**

A neonatal mannequin was ventilated with a set range of TVs (2–6 ml), different inspired fractional oxygen (FiO_2_) (0.21 and 1.00), and respiratory rate settings (10–60 breaths per minute), and GASMAS measurements were acquired.

**Results:**

For both FiO_2_ levels, the mean O_2_ projected concentration (PC) was significantly higher during inspiration compared to expiration for all TV values (*p* < 0.05). However, the difference in mean O_2_ PC between the inspiration and expiration phases depended on the TV (*p* < 0.001 for phase*TV interaction). The differences between the inspiration and expiration phases increased progressively with rising TV values. The oxygen absorption difference between inspiration and expiration differed by respiratory rate (*p* < 0.001).

**Conclusion:**

GASMAS detects the difference between inspiration, expiration, and tidal volume gas changes, suggesting a potential clinical application of GASMAS for respiratory monitoring of ventilated neonates.

**Impact:**

- What is the key message of your article?
- An experimental study demonstrating the feasibility of the GASMAS technique for detecting changes in lung gas volume.
- What does it add to the existing literature?
- GASMAS detects the difference between inspiratory and expiratory breath phases, various tidal volumes, oxygen concentration, and respiratory rate.
- What is the impact?
- This suggests a potential clinical application of GASMAS for respiratory monitoring of neonates.

## Introduction

In the last few decades, significant advances in ventilation strategies for preterm neonates have evolved, centered around targeted tidal volume (TV) ventilation and avoiding the negative aspects of ventilator-induced lung injury.^1^ Therefore, precise monitoring of TV administered to ventilated patients in neonatal intensive care units (NICUs) is essential but remains challenging. In neonates, TV is commonly measured using flow sensors located within the ventilator's respiratory circuit away from the patient. Flow sensor-based volume monitoring can be inaccurate for multiple reasons. Sensors may lack systemic accuracy and cause more or less of the volume delivered to the patients compared to the preset value.^2^ The actual gas volume reaching alveoli may be different from that measured by flow sensors due to endotracheal tube (ETT) leak, which results in underestimation of expired volume. Furthermore, some of the inspired volume detected has come from the anatomical dead space and does not represent gas from the respiratory bronchioles and alveoli. This dead space, which approximates 1.2–2.7 ml/kg, represents gas that remains inside the ETT and conductive airways (trachea and bronchi).^3^ It is generally larger in neonates than in adults, especially extremely low birth weight preterm babies,^4^ and neonates with respiratory distress syndrome or bronchopulmonary dysplasia.^3^

Current clinical monitoring techniques lack the capacity to provide accurate and dynamic measurements of alveolar TV, which would more precisely indicate breath-by-breath lung volume changes, facilitating optimal gas exchange.

Gas in Scattering Media Absorption Spectroscopy (GASMAS) enables non-invasive lung gas detection up to approximately 3 cm from the skin surface.^5^ In neonatal lungs, this depth mainly comprises condensed alveolar tissue rather than conductive airways. Moreover, the measurement of gas absorption magnitude and calculation of pathlength (the distance the light traveled through the tissues before reaching the detector) enables the determination of the probed lung gas volume underneath. Continuous GASMAS measurement with high sampling frequency would allow evaluation of volume changes during different phases of the breathing cycle. This could provide valuable information about physiological lung dynamics and lead to more accurate non-invasive monitoring of respiratory status to support gentle ventilation strategies while maintaining optimal TV. It may help to determine the effects of different invasive and non-invasive ventilation strategies in newborns. Broader potential clinical applications of GASMAS in neonatal respiratory care are described previously.^6^

The potential application of this optical technology to detect different lung gas volumes has been described in a few previous experimental, in vitro, and in vivo studies,^7–9^ but to our knowledge, dynamic TV changes have never been investigated in a simulated model of mechanical ventilation. We hypothesized that GASMAS can detect different TV delivered to the mechanically ventilated lungs of a neonatal mannequin model, breath-by-breath.

## Methods

### Gas in scattering media absorption spectroscopy

**Fig. 1:**
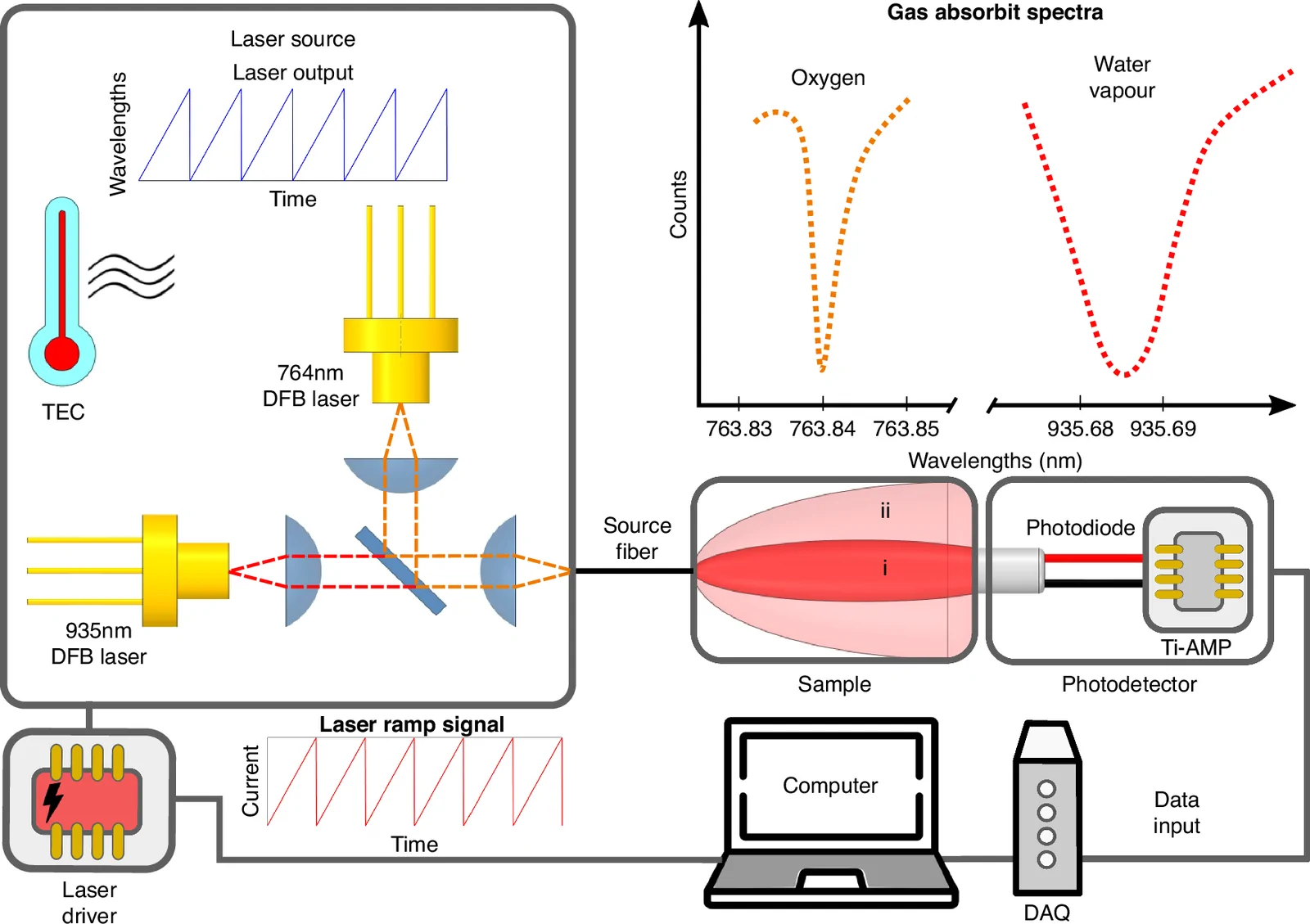
Schematic drawing of the GASMAS system and the characteristic gas absorption peaks for oxygen (O_2_) and water vapor (H_2_O) gases. TEC thermoelectric cooler, DFB Distributed FeedBack diode, i diffuse light path collected by photodiode, ii diffuse light not collected by photodiode, Ti-AMP transimpedance amplifier, DAQ data acquisition card.

GASMAS is an optical technology used to quantify gas concentration inside diffuse matter (e.g., lung tissue) by targeting very narrow energy absorption bands of molecular oxygen (O_2_) and water (H_2_O) vapor. A dual near-infrared light source at the wavelengths matching the characteristic gas absorption bands between 763–764 nm and 935–936 nm for O_2_ and H_2_O gases, respectively, is coupled into the transcutaneous probe (diffuser) and used to illuminate tissues of interest. As both laser sources are tunable and their respective emission wavelengths can be quickly increased or decreased, the gas absorption bands can be scanned to produce the absorption spectra. These spectra are then recorded by a photodetector as intensity versus wavelengths and contain the characteristic gas absorption peak (Fig. 1). The magnitude of this peak is then used to calculate gas concentration by means of the *Beer–Lambert law* applicable for gases, which states that the intensity of the emerging light (I), propagating through a gas with concentration ($c,\, \%$) and absorption cross-section (ε) along an optical pathlength ($l,{m}$), decays exponentially (Eq. 1).

1 $$I={I}_{0}{e}^{-\varepsilon {cl}}$$

The absorption signal of H_2_O vapor is required as a reference to calculate the optical pathlength (*l*), assuming it is equal for both gases (H_2_O vapor and O_2_). The concentration of H_2_O vapor in a cavity with 100% relative humidity and known gas temperature is derived from the *Ideal Gas Law* and the *Arden Buck equation*.^7,10^

To demonstrate the simultaneous contribution of light absorption and scattering effects on the measured signal within the biological tissue, the absorption magnitude is presented as projected concentration (*PC*, %m). This is a unit used in previous GASMAS studies^11,12^ and is dependent on the gas concentration (*c*, %) at known temperature and pressure of the gas and its mean optical pathlength (*l*, m) (Eq. 2), with both derived from Eq. 1.

2 $${PC}=c* l$$

For example, if O_2_ gas accounts for 20% of all the gases in the measured sample at known temperature and pressure, and the mean optical pathlength is approximately 5 cm (0.05 m), this would result in a PC of 20 × 0.05 = 1%m.

### Experimental setup

This experimental study was performed using a mannequin simulating a preterm neonate (Premature Ann®, Leardal) with anatomically proportional airways and two plastic bags attached to the trachea inside the chest simulating lungs. The mannequin’s total (maximal) lung volume capacity, as per the manufacturer, was 10 ml for each lung (20 ml in total), which represents the 50th percentile of fetal total lung volume at 24 weeks of gestation.^13^ The contents of the thoracic cavity of the mannequin included a layer of plastic foam between the lung and the rubber-based outer layer acting as a skin. A cross-sectional schematic description of the mannequin’s contents and changes of light travel path during inspiration and expiration is provided in Supplementary Fig. 1. Optical features of the mannequin’s materials were tested with time-of-flight absorption spectroscopy^14,15^ that showed similar absorption and scattering properties to human tissues, including skin, fat, muscle, lung, and bone (Table 1).^16–19^

**Table 1:** Optical properties (absorption and scattering) of materials of the mannequin at two wavelengths with time-of-flight absorption spectroscopy.

	Absorption (cm^−1^)	935 nm	Reduced scattering (cm^−1^)	935 nm
	764 nm		764 nm	
Materials of the mannequin				
All chest layers, left side	0.09	0.09	8.76	8.32
All chest layers, right side	0.09	0.09	7.53	8.13
Chest foam only, left side	0.05	0.05	18.63	16.39
Chest foam only, right side	0.06	0.07	17.41	16.71
Mean	0.07	0.08	13.08	12.39

The mannequin was connected to a neonatal ventilator (SLE5000, SLE) via an endotracheal tube (ETT) with an inner diameter of 2.5 mm. The correct ETT tip position was confirmed by auscultation with air entry audible bilaterally and absence of a leak on a ventilator display. The ETT was secured using medical tape at a depth of 6 cm, measured at the level of the lips. The mechanical ventilator was set to Synchronized Intermittent Mandatory Ventilation (SIMV) mode with targeted tidal volume (TV). The ventilator in this mode delivered a specified number of breaths each minute, with a preset inspiratory volume and duration. Ambient air was delivered to the ventilator by a standard hospital gas supply system. A heated humidifier device (MR850 Fisher & Paykel) was connected to the ventilator and humidified and heated the outlet gas to 100% and 37 °C, respectively.

**Fig. 2:**
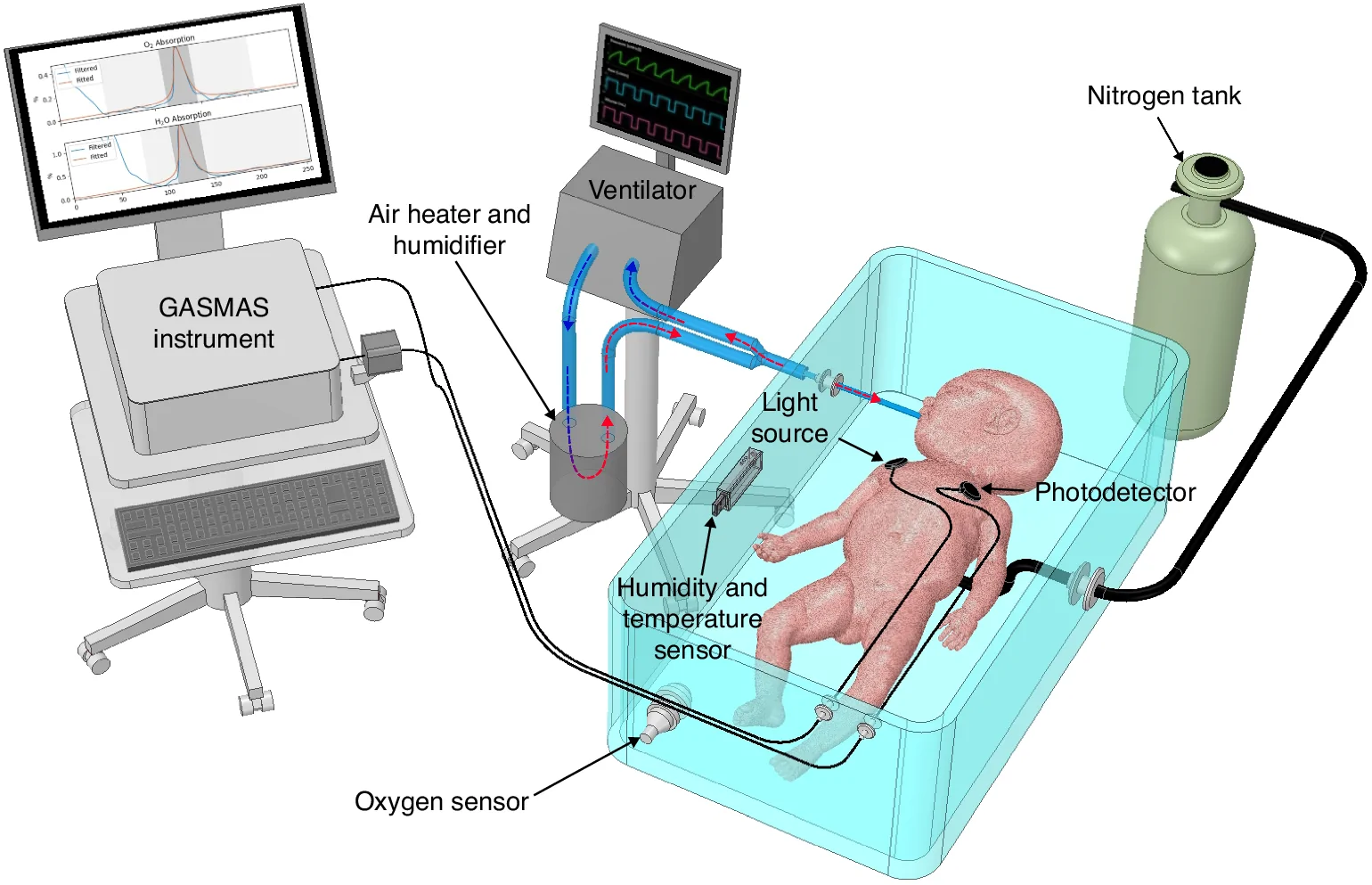
Schematic experimental setup. Ventilated neonatal mannequin with positioned GASMAS light source probe and photodetector, inside the box of a controlled environment. Images are not to scale.

The mannequin was placed inside the sealed plastic enclosure, which was purged with nitrogen gas overnight, to replace O_2_ and reduce humidity to 7.2% inside the enclosure and the foam surrounding the lungs of the mannequin’s chest. This particular enclosure was designed to replicate conditions within the neonatal chest, with the model’s foam representing the porous media. O_2_, humidity, and temperature sensors were used to indicate the level of nitrogen purging and to monitor general environment conditions of the enclosure (Fig. 2). The enclosure was covered with light-blocking fabric to reduce contamination by ambient light.

The GASMAS measurements were performed using a NEOLA Alpha 2 Lung Analyzer (NEOLA Medical) with two laser sources sweeping over the wavelengths of 763.83–763.85 nm and 935.68–935.69 nm and a sampling frequency of 4–5 Hz.

The performance of the GASMAS system was confirmed by obtaining a measurement on a reference gas cell with known conditions: 30 mm pathlength, 100% relative humidity, and ambient room temperature.

Then the laser source diffuser probe was placed onto a mannequin on the left frontal side of the chest above the internal lung. A photodetector probe, with a detection area of 1 × 1 cm, was positioned at a distance of 8 cm from the light source on the right side of the chest. To minimize the amount of ambient air present in the illumination path, a small quantity of ultrasound gel was applied between the source and detector probes and the skin of the mannequin.

A total of 16 GASMAS measurement sessions were conducted, each lasting 12.5 s. Within each session, 50 absorption spectra were acquired, resulting in a duration of 0.25 s per spectrum. The first five sessions were used to observe the instrument’s performance at different TVs of ventilation rates (2–6 ml in steps of 1 ml) with FiO_2_ of 0.21. The next five measurement sessions were repeated in the same configuration as above, only changing the FiO_2_ to 1.00. Throughout these ten sessions, the respiratory rate (RR) was set to 20 breath cycles per minute (3 s per breath, 2 s inspiration, and 1 s expiration). We chose different durations for inspiration and expiration to ensure the correct recognition of each phase. For each breath cycle within a session, the average H_2_O PC and average O_2_ PC were calculated for the inspiration phase and the expiration phase separately, and these averages were used in the statistical analysis. The last six measurement sessions were used to test the performance at different RRs (10–60 bpm in steps of 10) with varying inspiration and expiration times. For these sessions, TV and FiO_2_ were set to 5 ml and 1.00, respectively.

### Statistical analysis

Continuous variables are described using the mean and standard deviation (SD). To investigate if H_2_O PC and O_2_ PC differed between the inspiration phase and the expiration phase, and if that change depended on TV, a linear mixed model was used. The dependent variable in the model was H_2_O PC or O_2_ PC, and the fixed effects were TV (2 ml, 3 ml, 4 ml, 5 ml, 6 ml) and phase (inspiration, expiration), and the two-way interaction phase*TV. Phase and TV were treated as categorical variables in the model. A statistically significant interaction would indicate that the change in the dependent variable between the inspiration and the expiration phase differed by TV. Breath cycle number was included as a random effect in the model. Separate analyses were performed for each FiO_2_ level (0.21, 1.00). Using the same mixed models presented, pairwise comparisons were performed to investigate whether H_2_O PC or O_2_ PC differed by TV during both the inspiration and expiration phases. Pearson’s correlation coefficient was also used to measure the strength of the relationship between the change in H_2_O PC (or O_2_ PC) between the inspiration and expiration phases (inspiration phase – expiration phase) and TV value for each FiO_2_ level separately.

For both H_2_O PC and O_2_ PC, separate linear regression models were used to assess whether the relationship between differences in the inspiration and expiration phases and TV varied by FiO₂ level. The dependent variable in each model was the difference in H_2_O PC (or O_2_ PC) between the inspiration and expiration phases. The independent variables were TV (2 ml, 3 ml, 4 ml, 5 ml, 6 ml), FiO_2_ level (0.21, 1.00), and the interaction between TV and FiO_2_ level. A statistically significant interaction would indicate that the relationship between changes in the dependent variable and TV differs depending on the FiO_2_ level.

To investigate if O_2_ PC differed between the inspiration phase and the expiration phase and if that change depended on the RR, a linear mixed model was used. The dependent variable in the model was O_2_ PC, and the fixed effects were RR (10, 20, 30, 40, 50, 60 breaths per minute) and phase (inspiration, expiration), and the two-way interaction phase*RR. Phase and RR were treated as categorical variables in the model. A statistically significant interaction would indicate that the change in the dependent variable between the inspiration and the expiration phase differed by RR. Breath cycle number was included as a random effect in the model.

All tests were two-sided, and a *p*-value < 0.05 was considered statistically significant. Statistical analysis was performed using IBM SPSS Statistics (Version 27, IBM Corp., Armonk).

## Results

### Water vapor absorption (H_2_O PC) with different respiratory phases, tidal volumes, and inspired fractional oxygen

**Fig. 3:**
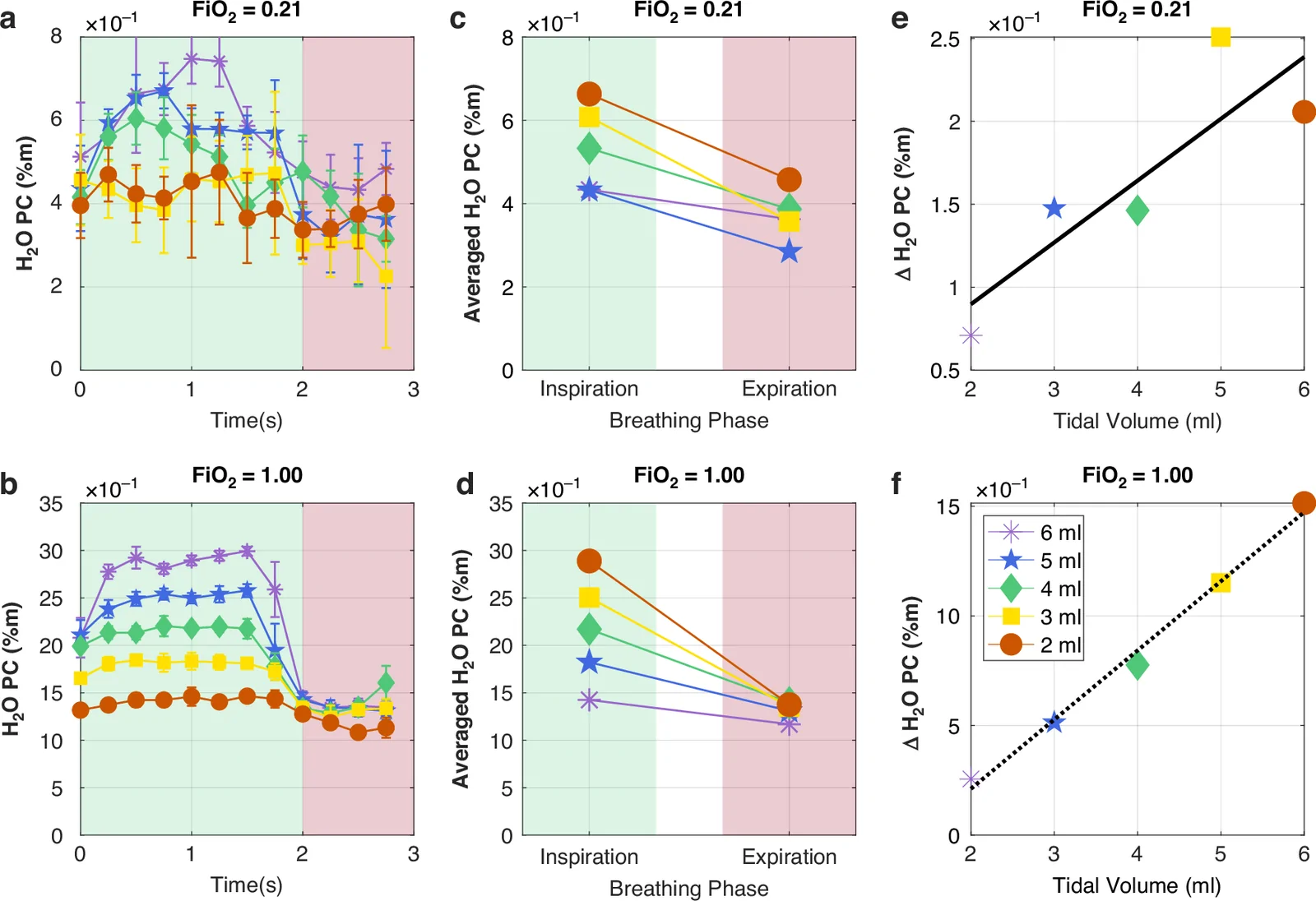
Water vapor absorption (H_2_O PC) across different respiratory phases, tidal volume, and inspired fraction of oxygen. **a** and **d** H_2_O PC (mean, SD) for a range of different tidal volumes (2–6 ml) (see legend) over time (breath phases) and FiO_2_ (0.21 – first row, 1.00 – second row). Each data point represents the average value of the four breath cycles. **b** and **e** Estimated marginal means for H_2_O PC for inspiration vs expiration and FiO_2_ (0.21 – first row, 1.00 – second row). The respiratory rate was set to deliver 20 breaths per minute with the duration of the inspiration phase of 2 s (shaded in green) and expiration – 1 s (shaded in red). **c** and **f** The difference (Δ) of H_2_O PC between the two breath phases (inspiration and expiration) at the two FiO_2_ values (legend) as a function of the tidal volume. PC projected concentration, FiO_2_ fraction of inspired oxygen, H_2_O water vapor, O_2_ oxygen gas.

H_2_O vapor PC values across the different respiratory phases, TV, and FiO_2_, are presented in Fig. 3.

For each FiO_2_ value, differences in mean (∆) H_2_O PC between the inspiration and expiration phase depended on TV (*p* < 0.001 for phase*TV interaction) (Supplementary Table 1, Fig. 3c, f). For all TV values (except TV = 2 ml when FiO_2_ = 0.21), mean H_2_O PC was significantly higher during inspiration compared to expiration (Fig. 3b, e), and differences increased with increasing TV for both FiO_2_ values (Supplementary Table 2).

During inspiration, increasing gas TV resulted in incrementally increasing magnitude of H_2_O vapor absorption (Fig. 3b, e). For each FiO_2_ value, all pairwise comparisons between TV groups were statistically significant (*p* < 0.001), indicating that the mean H_2_O PC during the inspiration phase was influenced by the TV (Supplementary Table 3). During expiration at FiO_2_ of 0.21, mean H_2_O PC was significantly different for each TV group comparison (*p* < 0.05), except when comparing mean H_2_O PC for 4 and 2 ml (*p* = 0.33). During expiration at FiO_2_ of 1.00, mean H_2_O PC was significantly higher for TV of 6 ml compared to all other TV values (*p* < 0.001). No other pairwise comparisons were statistically significant (Supplementary Table 3).

There was a strong positive correlation between the difference in H_2_O PC values (inspiration-expiration) and TV values (*r* = 0.96; *p* < 0.001) at FiO_2_ 0.21, and at FiO_2_ 1.00 (*r* = 0.98; *p* < 0.001) (Fig. 3c, f). The relationship between the change of H_2_O PC values (inspiration-expiration) and TV was not significantly affected by FiO_2_ level (*p* = 0.21 for TV*FiO_2_ interaction).

### Oxygen gas absorption (O_2_ PC) with different respiratory phases, tidal volumes, and inspired fractional oxygen

**Fig. 4:**
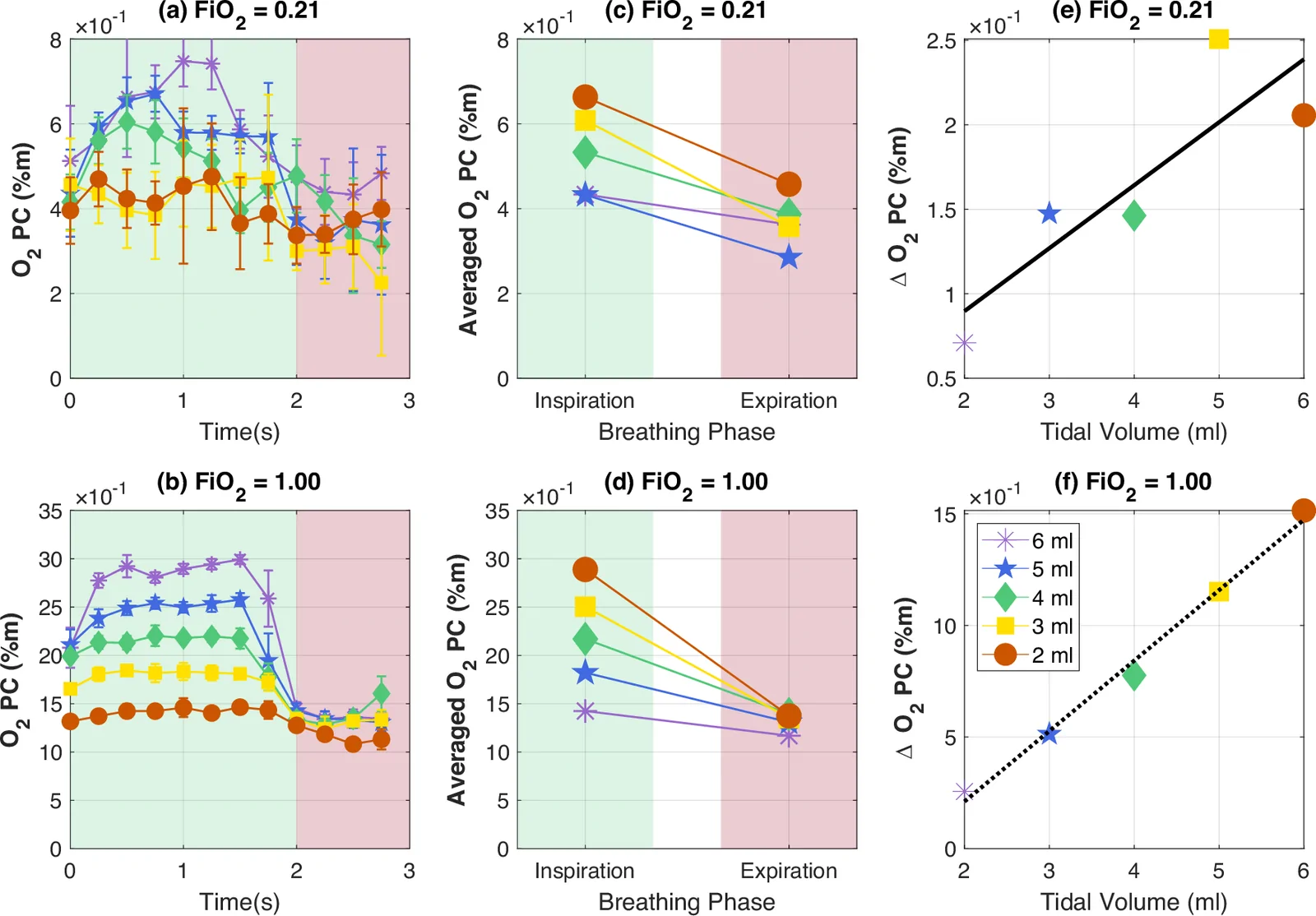
Oxygen gas absorption (O_2_ PC) across different respiratory phases, tidal volume, and inspired fractional oxygen. **a** and **d** O_2_ PC (mean, SD) for a range of different tidal volumes (2–6 ml) (see legend) over time (breath phases) and FiO_2_ (0.21 – first row, 1.00 – second row). Each data point represents the average value of the four breath cycles. **b** and **e** Estimated marginal means for O_2_ PC for inspiration vs expiration and FiO_2_ (0.21 – first row, 1.00 – second row). The respiratory rate was set to deliver 20 breaths per minute with the duration of the inspiration phase of 2 s (shaded in green) and the expiration – 1 s (shaded in red). **c** and **f** The difference (Δ) of O_2_ PC between the two breath phases (inspiration and expiration) at the two FiO_2_ values (0.21 – first row, 1.00 – second row) as a function of the tidal volume. PC projected concentration, FiO_2_ fraction of inspired oxygen, H_2_O water vapor, O_2_ oxygen gas.

O_2_ PC values across the different respiratory phases, TV, and FiO_2_, are presented in Fig. 4.

For each FiO_2_ value, differences in mean (∆) O_2_ PC between the inspiration and expiration phase depended on TV (*p* < 0.001 for phase*TV interaction) (Supplementary Table 4; Fig. 4c, f).

At a FiO_2_ value of 0.21, the mean O_2_ PC was significantly higher at inspiration compared to expiration for all TV values (*p* < 0.05 for all, Supplementary Table 5; Fig. 4b) and the largest difference was observed between the inspiration and expiration phases when TV = 5 ml (difference in means (95% CI): −0.25 (−0.30 to −0.20), *p* < 0.001).

At a FiO_2_ value of 1.00, the mean O_2_ PC was also significantly higher at inspiration compared to expiration for all TV values (*p* < 0.001), and differences between the inspiration and expiration phase increased gradually with increasing TV (Supplementary Table 5; Fig. 4e).

During inspiration at FiO_2_ of 0.21, no significant differences in the magnitude of O_2_ gas absorption were found when the TV was 2 versus 3 ml (marginal mean = 0.43 for both, *p* = 0.98). Although at higher levels of TV, increasing gas TV resulted in a gradually increasing magnitude of O_2_ gas absorption (Supplementary Table 6, Fig. 4b). During inspiration at FiO_2_ of 1.00, increasing gas TV also resulted in a gradually increasing magnitude of O_2_ gas absorption (Supplementary Table 6, Fig. 4e).

For each FiO_2_ value (except TV = 3 ml vs TV = 2 ml at FiO_2_ 0.21, *p* = 0.98), all pairwise comparisons between TV groups were statistically significant (*p* < 0.05), indicating that the mean O_2_ absorption during the inspiration phase was influenced by the TV (Supplementary Table 6).

During expiration at FiO_2_ of 0.21, mean O_2_ absorption was significantly higher for TV of 6 ml compared to all other TV values and significantly lower for TV of 3 ml compared to all other TVs. No significant differences were found in mean O_2_ absorption when TV = 2, 4, or 5 ml. During expiration at FiO_2_ of 1.00, mean O_2_ was significantly lower for TV of 2 ml compared to all other TV values, and significantly lower for TV of 3 ml compared to TV of 4 and 6 ml. No other pairwise comparisons were statistically significant (Supplementary Table 6).

There was a strong positive correlation between the difference in O_2_ PC values (Δ = inspiration − expiration) and TV values (*r* = 0.72; *p* < 0.001) at FiO_2_ 0.21 and FiO_2_ 1.00 (*r* = 0.99; *p* < 0.001). (Fig. 4c, f). The relationship between the change of O_2_ PC values (inspiration-expiration) and TV was statistically significant (*p* < 0.001 for TV* FiO_2_ interaction), indicating that the relationship between the change in the mean PC and TV differed by FiO_2_ level, with larger increases in difference between inspiration and expiration for a 1 ml increase in TV when FiO_2_ 1.00 compared to when FiO_2_ 0.21.

### Oxygen gas absorption with different respiratory rates

In all measurement sessions performed with different respiratory rates (RR), we observe O_2_ PC corresponding to different breathing phases by increasing with inspiration and decreasing with expiration despite changing the duration of inspiration/expiration with set RR (Supplementary Fig. 2). The difference in mean O_2_ PC between the inspiration and expiration phase depended on the RR (*p* < 0.001 for phase*RR interaction) (Supplementary Fig. 2 and Supplementary Table 7). For all set RR values, mean O_2_ PC was significantly higher (*p* < 0.001 for all) at inspiration compared to expiration (Supplementary Fig. 2 and Supplementary Table 8).

## Discussion

This is the first experimental study investigating the relationship between molecular O_2_ gas absorption by specific near-infrared light and gas volume changes during simulated ventilator-delivered breaths in a preterm neonatal mannequin model. The neonatal mannequin’s chest rise was observed with each inspiration and chest fall with each expiration, and corresponded to both the ventilator settings and the real-time assessment by the GASMAS system. This observation confirmed the operational integrity of the experimental model. While GASMAS does not directly provide gas volume measurements, it yields information on gas absorption magnitude within the lungs, which depends on the volume and concentration of gas being probed. Our findings have demonstrated that H_2_O and O_2_ absorption magnitude increase with inspiration and decrease with expiration. Subsequently, and of greater clinical relevance, GASMAS was evaluated for its ability to detect changes specifically related to TV. For that, the gas absorption values provided by the GASMAS were compared during the breaths delivering different TVs. It is important to note that TVs were measured using a ventilator flow sensor (located within the respiratory circuit) while GASMAS was used as a proxy to assess whether the administered TV was reflected within the mannequin’s lungs.

Our findings have demonstrated that H_2_O and O_2_ absorption magnitude incrementally increase with larger TVs delivered in a neonatal mannequin model, and each additional 1 ml increase in TV was associated with significant O_2_ and H_2_O PC change. Two exceptions were observed: (i) comparisons of H₂O PC at baseline residual lung volume (expiration) with H_2_O PC at a TV of 2 ml, and (ii) comparisons of O_2_ PC between 2 and 3 ml at an FiO₂ of 0.21, both of which did not reach statistical significance. These exceptions highlight the importance of the total probed lung volume. Specifically, the absorption signal improves as the probed gas volume increases, with O_2_ absorption showing a more pronounced dependence due to its inherently weaker spectral signal.

These results demonstrate proof-of-concept sensitivity of GASMAS to TV ranging from 2 to 6 ml, supporting the feasibility of using GASMAS for breathing assessment in preterm infants, given that the typical TV is 4–6 ml/kg [2] (4–6 ml for a 1 kg neonate; 2–3 ml for a 0.5 kg neonate). It is uncertain whether TV variations of less than 1 ml exert a measurable influence on absorption parameters, which has been demonstrated previously by Pacheco et al.^7^ Further studies are required to calibrate signal magnitude against absolute TV to establish clinical thresholds and evaluate the agreement between ventilator-measured TVs and gas volume changes detected with GASMAS in neonatal patients by synchronizing data acquisition from both devices.

Secondly, the question of how this technique should be calibrated to account for differences in lung volume (tidal and residual) based on neonatal body weight, if measurements are performed on different-sized patients varying with gestational age, remains to be determined. The clinical observational study led by a research group from the Infant Research Centre at University College Cork and Tyndall Institute has performed GASMAS lung measurements, including different weight term neonates, showing no variations of measurements in relation to weight.^20^ Future investigations should incorporate measurements across low, very low, and extremely low birth weight patient categories to address inter-subject variability.

O_2_ absorption changes to a stepwise titration of FiO_2_ were demonstrated in a previous study with piglets ventilated using a constant TV.^8^ Our findings also support the statement that GASMAS measured O_2_ signal depends on gas concentration (FiO_2_) as well as probed volume. This presents a challenge within the clinical setting where ventilated neonates often require both – adequate target TV and additional FiO_2_ ranging from 0.21 to 1.0 to maintain sufficient gas exchange and blood O_2_ saturation. The effects of TV and FiO_2_ on absorption were distinct: O_2_ PC values increased fourfold when FiO_2_ changed from 0.21 to 1.0, while increasing TV by 1 ml, raised O_2_ PC proportionally by 1.1–1.2 times despite different FiO_2_ (Fig. 4a, d). These findings should be incorporated into future GASMAS signal processing algorithms to account for different O_2_ fractions probed within the lung to distinguish the signal variation due to gas concentration.

Low sensitivity with the standard deviation ranges of O_2_ PC overlapping at FiO_2_ of 0.21 (Fig. 4a), was noted as well as more differentiated absorption values at FiO_2_ of 1.00 (Fig. 4d), suggesting higher O_2_ concentration causes more pronounced absorption. Higher GASMAS sensitivity in neonates, especially at lower FiO_2,_ may be achieved by improving signal-enhancing techniques and by placing the probes in transmittance geometry with an intratracheal light source, as demonstrated using the photon hitting density modeling by Pacheco et al.^9^

In this study, we employed a single anterior placement of both the light source and detector probe. Based on the principle of the “banana-shaped” path of light propagation,^21^ this configuration provides information on gas absorption beneath the probes, thereby enabling the detection of localized gas volumes within specific regions of the lung.^22,23^ Multisite placement would allow for comprehensive mapping of pulmonary regions, thereby offering a representation of overall gas distribution, which may enhance detection of TV variations due to atelectasis, hyperinflation, air trapping, pneumothorax, and direct therapies to improve gas exchange. This approach should be considered in the refinement of future technological development and the design of subsequent research studies.

There are some technical differences between the respiratory model we used and neonatal chest that should be noted when considering the comparability of both. The temperature and humidity controls, along with nitrogen purging, were only required for the mannequin experiments, as certain regions were open to airflow and could contain gases in locations where they would not be present in vivo. Therefore, such calibration is not needed for clinical scenarios, as the neonatal chest cavity is composed of gas, delivered by the ventilator after being humidified and heated. When the probes are well secured to the chest, there should be no environmental (gas surrounding the neonate inside of incubator) interference.

Furthermore, the respiratory model used in this experiment holds considerably low residual gas volume (maximal volume capacity – 10 ml per lung), potentially causing high absorption signal variation during the expiration phase. On the contrary, the healthy infants’ lung functional residual capacity accounts for approximately 30–45 ml for 1.5 kg, 20–30 ml for 1 kg, and 10–15 ml for 0.5 kg neonate. Therefore, measurements performed over the neonatal chest should carry a stronger absorption signal and provide more reliable information on gas volume probed during exhalation compared to the model used in this study.

The set of measurements presented in Supplementary Fig. 2 had noted variation of O_2_ PC averaged values during inspiration and expiration phases, dependent on the underlying RR. O_2_ PC inspiratory and expiratory values were higher compared to corresponding breath phases at elevated RRs. This is likely related to the features of the model used, causing progressive gas volume accumulation within the lungs during short expiration in the absence of the appropriate recoil force of the mannequin’s chest wall. Additional variation could be owing to instrumental inaccuracies that can cause the ventilator to deliver up to ±10% difference in set breaths per minute.^2^

Svanberg et al. noted the O_2_ absorption variation corresponding to breath phases^8^, which we have explored in more detail using different durations of inspiration and expiration (Supplementary Fig. 2). The absorption values correlated with gas volume changes of different breath phases, but some of the measurements performed showed the potential limits of the GASMAS technique due to acquisition duration. The minimum acquisition time (or time responsiveness) of the GASMAS integrated analyzer currently is 0.25 s, which means it provides one data point for the measurement performed over 0.25 s. To maximize signal quality with the current 0.25 s acquisition window, slow respiratory rates and prolonged inspiratory time (Ti) (0.75–2 s) were selected in this study. It has allowed us to demonstrate, for the first time, that the breath cycle can be monitored using GASMAS-derived gas signals alone. We acknowledge that the present acquisition speed limits applicability under typical neonatal ventilator settings (Ti 0.3–0.4 s^24^), where short Ti may introduce signal aliasing. Future work should address this through (i) synchronous ventilator–GASMAS triggering with multi-phase sampling to enable alias-free reconstruction of the breath waveform, and (ii) improved optical throughput—via enhanced light coupling and a wider-area detector—to increase signal strength and reduce acquisition time to approximately 75 ms.

To obtain GASMAS measurements, a few theoretical assumptions are used, including that the pathlength for the two different wavelengths (for O_2_ and H_2_O) is the same. In a practical setting, it is important to acknowledge that this assumption may not be fully justified, potentially leading to an over-estimation of O_2_ absorption. Further studies focusing on modeling the pathlengths of the two wavelengths are required to investigate the differences and find correction factors to obtain more accurate O_2_ absorption measures.

Overall, using GASMAS, we were able to demonstrate dynamic gas absorption measurements of different TVs delivered, representing gas volume changes breath-by-breath during inspiration and expiration, supporting our hypothesis.

The continuity of technical advancements in line with the future observations from experimental and clinical studies in collaboration with biophotonic engineers and scientists, clinical researchers, clinicians, university and industry partners has a major role in supporting the translation of innovative GASMAS technology into a clinically applicable, non-invasive system for neonatal respiratory monitoring by addressing important challenges for effective implementation.^25^

## Conclusions

This is the first study that demonstrates the feasibility of GASMAS as a technique for oxygen and lung gas volume quantification in a dynamic setting. We showed that in a simulated preterm infant lung model, the GASMAS technique can be utilized to detect changes in tidal volume, inspiratory and expiratory phases of respiration, respiratory rates, and oxygen concentration. GASMAS has potential clinical application for more accurate non-invasive respiratory monitoring of ventilated neonates.

## Supplementary information

Supplementary Figure 1

Supplementary Figure 2

Supplementary Tables 1-8

## Data Availability

Data underlying the results presented in this paper are available in the Denodo repository: Fig. 3 and 4 at https://zenodo.org/records/14918521 and Supplementary Fig. 2 at https://zenodo.org/records/13838914.
