# Supplementary material for: Breath-by-breath lung gas volume detection using GASMAS in a neonatal mannequin
Source: Pediatr Res. 2026 Jan 10;100(3):1440–7. doi: 10.1038/s41390-025-04699-2 (PMC13672044; doi:10.1038/s41390-025-04699-2)
Supplement: Supplementary file 1 [file 41390_2025_4699_MOESM1_ESM.pdf]

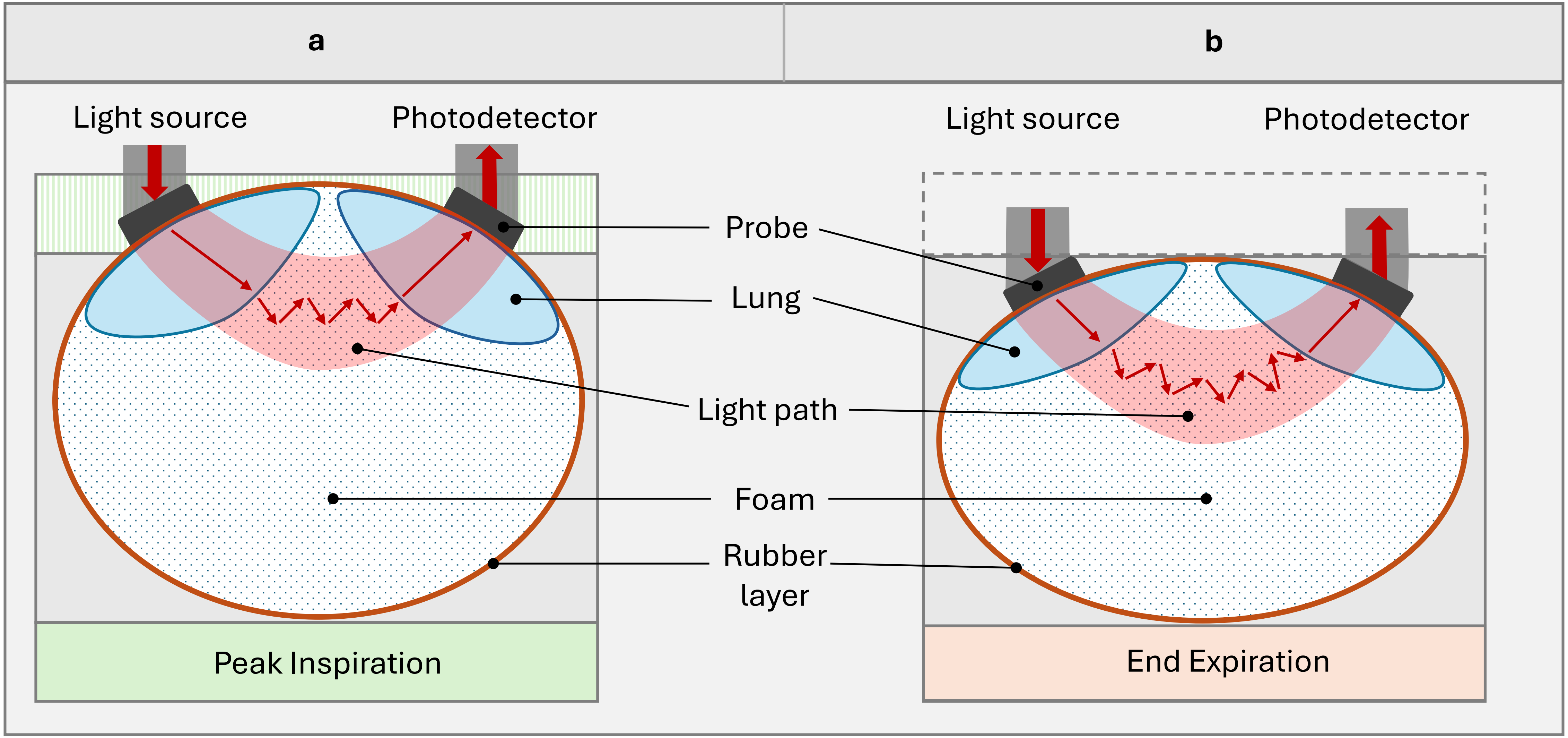

Supplementary Figure 1. A cross-section schematic description of the mannequin's contents and changes of light travel path during breath phases (a – peak inspiration, b – end expiration).
