# Supplementary material for: Breath-by-breath lung gas volume detection using GASMAS in a neonatal mannequin
Source: Pediatr Res. 2026 Jan 10;100(3):1440–7. doi: 10.1038/s41390-025-04699-2 (PMC13672044; doi:10.1038/s41390-025-04699-2)
Supplement: Supplementary file 2 [file 41390_2025_4699_MOESM2_ESM.pdf]

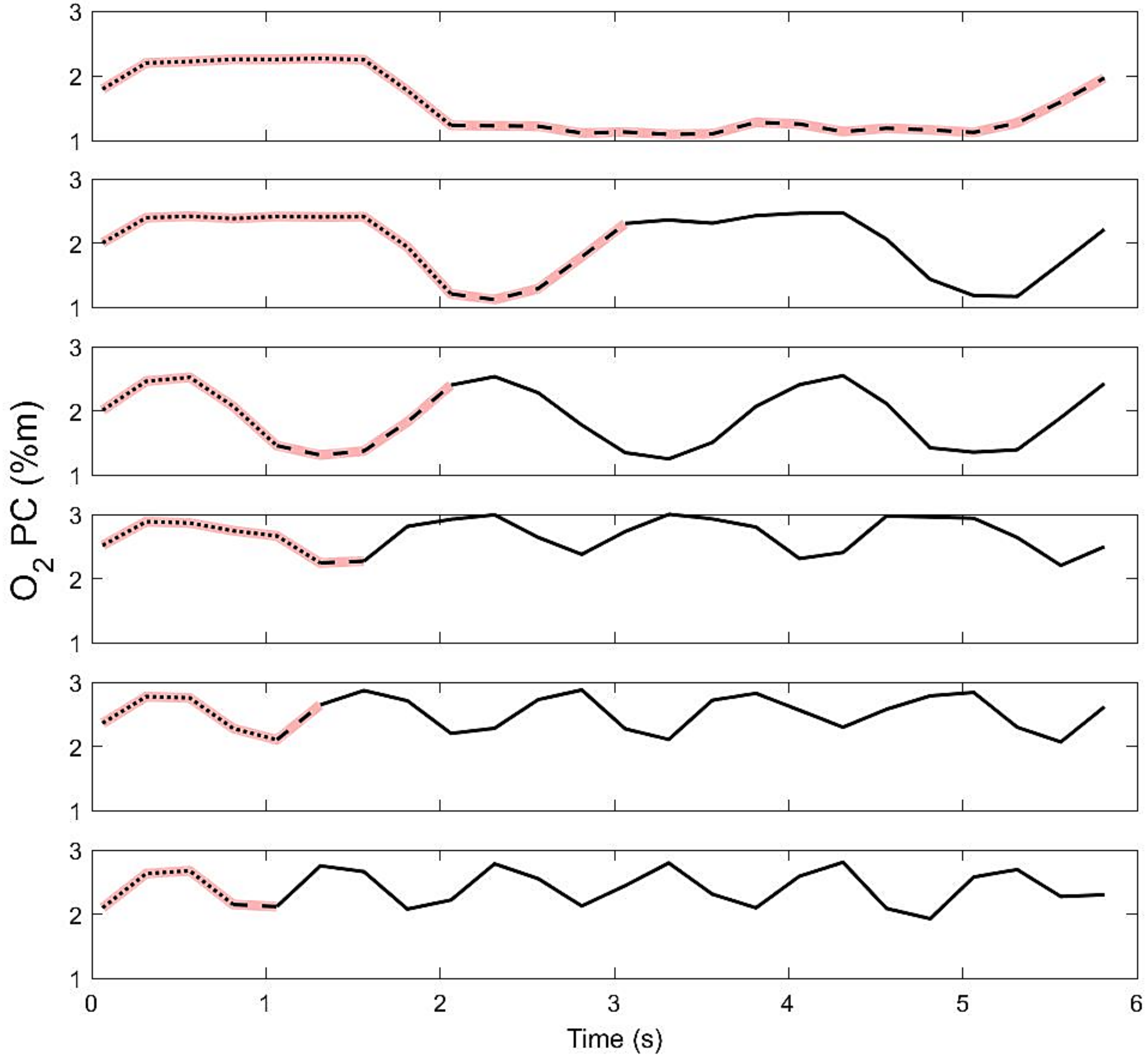

Respiratory Rate, bpm	Breath Cycle, s	Inspiration, s	Expiration, s	# of Cycles shown
10	6	2	4	1
20	3	2	1	2
30	2	1	1	3
40	1.5	1.25	0.25	4
50	1.2	0.95	0.25	5
60	1	0.75	0.25	6

Supplementary Figure 2. Time plots of O2 PC for the six measurement sessions with different respiratory rates (RR, number of breaths per minute). The red shaded region in each of the subplots represents one breath cycle with dotted and dashed lines representing the inspiration and expiration phase of that breath cycle respectively. Although all the sessions lasted 12.5 s the results presented in the figure are averaged and truncated to avoid redundancy.
