# Supplementary material for: Breath-by-breath lung gas volume detection using GASMAS in a neonatal mannequin
Source: Pediatr Res. 2026 Jan 10;100(3):1440–7. doi: 10.1038/s41390-025-04699-2 (PMC13672044; doi:10.1038/s41390-025-04699-2)
Supplement: Supplementary file 3 [file 41390_2025_4699_MOESM3_ESM.pdf]

Supplementary Table 1. Results of linear mixed models for H_2O PC*

	FiO_2=0.21			FiO_2=1.00		
	coefficient	(95% CI)	p-value	coefficient	(95% CI)	p-value
Intercept	0.607	(0.591 to 0.624)	<0.001	0.798	(0.783 to 0.812)	<0.001
Phase			0.100			<0.001
Inspiration (ref)	0			0		
Expiration	-0.019	(-0.042 to 0.004)		-0.046	(-0.067 to -0.025)	
Tidal volume			<0.001			<0.001
2ml (ref)	0			0		
3ml	0.055	(0.032 to 0.078)		0.058	(0.037 to 0.079)	
4ml	0.131	(0.108 to 0.154)		0.113	(0.093 to 0.134)	
5ml	0.246	(0.223 to 0.269)		0.199	(0.178 to 0.220)	
6ml	0.350	(0.327 to 0.373)		0.315	(0.294 to 0.335)	
Phase*Tidal volume			<0.001			<0.001
Expiration*TV=3ml	-0.078	(-0.111 to -0.046)		-0.064	(-0.093 to -0.035)	
Expiration*TV=4ml	-0.119	(-0.152 to -0.087)		-0.109	(-0.138 to -0.080)	
Expiration*TV=5ml	-0.200	(-0.233 to -0.167)		-0.196	(-0.225 to -0.166)	
Expiration*TV=6ml	-0.230	(-0.262 to -0.197)		-0.258	(-0.288 to -0.229)	

*values of H_2O PC are 10^{-1}

Supplementary Table 2. Comparisons between the inspiration and expiration phases based on the linear mixed models for H_2O PC*

	FiO_2=0.21			FiO_2=1.00		
	difference in means**	(95% CI)	p-value	difference in means**	(95% CI)	p-value
Tidal volume						
2ml	-0.019	(-0.042 to 0.004)	0.100	-0.046	(-0.067 to -0.025)	<0.001
3ml	-0.098	(-0.121 to -0.074)	<0.001	-0.110	(-0.131 to -0.090)	<0.001
4ml	-0.138	(-0.161 to -0.115)	<0.001	-0.155	(-0.176 to -0.134)	<0.001
5ml	-0.219	(-0.242 to -0.196)	<0.001	-0.242	(-0.262 to -0.221)	<0.001
6ml	-0.249	(-0.272 to -0.226)	<0.001	-0.305	(-0.325 to -0.284)	<0.001

*values of H_2O PC are 10^{-1}

**expiration phase - inspiration phase

Supplementary Table 3. Estimated marginal means for each phase, TV value and FiO_2 value based on the linear mixed models for H_2O PC*

	marginal mean	(95% CI)	pairwise comparisons			
			vs 3	vs 4	vs 5	vs 6
<u>**FiO_2=0.21**</u>						
**Inspiration phase**						
Tidal volume						
2ml	0.607	(0.591 - 0.624)	<0.001	<0.001	<0.001	<0.001
3ml	0.662	(0.646 - 0.678)		<0.001	<0.001	<0.001
4ml	0.739	(0.722 - 0.755)			<0.001	<0.001
5ml	0.854	(0.837 - 0.870)				<0.001
6ml	0.958	(0.942 - 0.974)				
**Expiration phase**						
Tidal volume						
2ml	0.588	(0.572 - 0.605)	0.044	0.297	<0.001	<0.001
3ml	0.565	(0.548 - 0.581)		0.004	<0.001	<0.001
4ml	0.600	(0.584 - 0.617)			0.005	<0.001
5ml	0.634	(0.618 - 0.651)				<0.001
6ml	0.709	(0.693 - 0.725)				
<u>**FiO_2=1.00**</u>						
**Inspiration phase**						
Tidal volume						
2ml	0.798	(0.783 - 0.812)	<0.001	<0.001	<0.001	<0.001
3ml	0.856	(0.841 - 0.871)		<0.001	<0.001	<0.001
4ml	0.911	(0.896 - 0.926)			<0.001	<0.001
5ml	0.997	(0.982 - 1.012)				<0.001
6ml	1.112	(1.097 - 1.127)				
**Expiration phase**						
Tidal volume						
2ml	0.751	(0.736 - 0.766)	0.555	0.652	0.730	<0.001
3ml	0.745	(0.730 - 0.760)		0.301	0.352	<0.001
4ml	0.756	(0.741 - 0.771)			0.915	<0.001
5ml	0.755	(0.740 - 0.770)				<0.001
6ml	0.808	(0.793 - 0.823)				

*values of H_2O PC are 10^{-1}

Supplementary Table 4. Results of linear mixed models for O_2 PC

	FiO_2=0.21			FiO_2=1.00		
	coefficient	(95% CI)	p-value	coefficient	(95% CI)	p-value
Intercept	0.433	(0.395 to 0.472)	<0.001	1.425	(1.386 to 1.464)	<0.001
Phase			0.008			<0.001
Inspiration (ref)	0			0		
Expiration	-0.071	(-0.122 to -0.020)		-0.256	(-0.310 to -0.202)	
Tidal volume			<0.001			<0.001
2ml (ref)	0			0		
3ml	-0.001	(-0.051 to 0.050)		0.397	(0.343 to 0.452)	
4ml	0.099	(0.049 to 0.150)		0.746	(0.692 to 0.800)	
5ml	0.175	(0.124 to 0.225)		1.080	(1.026 to 1.134)	
6ml	0.230	(0.179 to 0.280)		1.465	(1.411 to 1.519)	
Phase*Tidal volume			<0.001			<0.001
Expiration*TV=3ml	-0.077	(-0.148 to -0.005)		-0.258	(-0.334 to -0.181)	
Expiration*TV=4ml	-0.075	(-0.147 to -0.004)		-0.520	(-0.597 to -0.444)	
Expiration*TV=5ml	-0.180	(-0.251 to -0.108)		-0.896	(-0.972 to -0.819)	
Expiration*TV=6ml	-0.135	(-0.206 to -0.063)		-1.259	(-1.335 to -1.182)	

Supplementary Table 5. Comparisons between the inspiration and expiration phases based on the linear mixed models for O_2 PC

	FiO$_2$=0.21			FiO$_2$=1.00		
	difference in means**	(95% CI)	p-value	difference in means**	(95% CI)	p-value
Tidal volume						
2ml	-0.071	(-0.122 to -0.020)	0.008	-0.256	(-0.310 to -0.202)	<0.001
3ml	-0.148	(-0.198 to -0.097)	<0.001	-0.514	(-0.568 to -0.459)	<0.001
4ml	-0.146	(-0.197 to -0.096)	<0.001	-0.776	(-0.830 to -0.722)	<0.001
5ml	-0.251	(-0.301 to -0.200)	<0.001	-1.152	(-1.206 to -1.097)	<0.001
6ml	-0.206	(-0.256 to -0.155)	<0.001	-1.515	(-1.569 to -1.461)	<0.001

**expiration phase - inspiration phase

Supplementary Table 6. Estimated marginal means for each phase, TV value and FiO_2 value based on the linear mixed models for O_2PC

	marginal mean	(95% CI)	pairwise comparisons			
			vs 3	vs 4	vs 5	vs 6
**FiO_2=0.21**						
**Inspiration phase**						
Tidal volume						
2ml	0.433	(0.395 - 0.472)	0.975	<0.001	<0.001	<0.001
3ml	0.433	(0.394 - 0.471)		<0.001	<0.001	<0.001
4ml	0.533	(0.494 - 0.571)			0.005	<0.001
5ml	0.608	(0.570 - 0.646)				0.034
6ml	0.663	(0.625 - 0.702)				
**Expiration phase**						
Tidal volume						
2ml	0.362	(0.324 - 0.401)	0.004	0.336	0.835	0.001
3ml	0.285	(0.247 - 0.324)		<0.001	0.007	<0.001
4ml	0.387	(0.348 - 0.425)			0.245	0.008
5ml	0.357	(0.319 - 0.396)				<0.001
6ml	0.458	(0.419 - 0.496)				
**FiO_2=1.00**						
**Inspiration phase**						
Tidal volume						
2ml	1.425	(1.386 - 1.464)	<0.001	<0.001	<0.001	<0.001
3ml	1.822	(1.783 - 1.861)		<0.001	<0.001	<0.001
4ml	2.170	(2.131 - 2.210)			<0.001	<0.001
5ml	2.505	(2.466 - 2.544)				<0.001
6ml	2.890	(2.851 - 2.929)				
**Expiration phase**						
Tidal volume						
2ml	1.169	(1.130 - 1.208)	<0.001	<0.001	<0.001	<0.001
3ml	1.309	(1.270 - 1.348)		0.003	0.102	0.018
4ml	1.394	(1.355 - 1.433)			0.133	0.476
5ml	1.353	(1.314 - 1.392)				0.417
6ml	1.375	(1.336 - 1.414)				

Supplementary Table 7. Results of linear mixed model for O_2 PC

	coefficient	(95% CI)	p-value
Intercept	1.992	(1.901 to 2.083)	<0.001
Phase			<0.001
Inspiration (ref)	0		
Expiration	-0.795	(-0.897 to -0.692)	
Respiratory rate (breaths per minute)			<0.001
10 (ref)	0		
20	0.379	(0.265 to 0.492)	
30	0.453	(0.333 to 0.574)	
40	0.896	(0.785 to 1.008)	
50	0.725	(0.617 to 0.834)	
60	0.787	(0.674 to 0.900)	
Phase*Respiratory rate			<0.001
Expiration*RR=10 (ref)	0		
Expiration*RR=20	-0.325	(-0.471 to -0.178)	
Expiration*RR=30	-0.321	(-0.468 to -0.173)	
Expiration*RR=40	-0.143	(-0.311 to 0.026)	
Expiration*RR=50	-0.127	(-0.294 to 0.040)	
Expiration*RR=60	-0.239	(-0.398 to -0.081)	

Supplementary Table 8. Comparisons between the inspiration and expiration phases based on the linear mixed model for O_2 PC

	difference in means*	(95% CI)	p-value
Respiratory rate			
(breaths per minute)			
10	-0.795	(-0.897 to -0.692)	<0.001
20	-1.120	(-1.224 to -1.015)	<0.001
30	-1.116	(-1.221 to -1.010)	<0.001
40	-0.937	(-1.072 to -0.803)	<0.001
50	-0.922	(-1.054 to -0.790)	<0.001
60	-1.034	(-1.155 to -0.913)	<0.001

*expiration phase - inspiration phase
